# Immune Cells as Critical Regulators of Steroidogenesis in the Testis and Beyond

**DOI:** 10.3389/fendo.2022.894437

**Published:** 2022-04-28

**Authors:** Xiaowei Gu, Shu-Yun Li, Satoko Matsuyama, Tony DeFalco

**Affiliations:** ^1^ Division of Reproductive Sciences, Cincinnati Children’s Hospital Medical Center, Cincinnati, OH, United States; ^2^ Department of Pediatrics, University of Cincinnati College of Medicine, Cincinnati, OH, United States

**Keywords:** Leydig cell, macrophage, steroidogenesis, testosterone, testis, immune cell, reproduction

## Abstract

Steroidogenesis is an essential biological process for embryonic development, reproduction, and adult health. While specific glandular cells, such as Leydig cells in the testis, are traditionally known to be the principal players in steroid hormone production, there are other cell types that contribute to the process of steroidogenesis. In particular, immune cells are often an important component of the cellular niche that is required for the production of steroid hormones. For several decades, studies have reported that testicular macrophages and Leydig cells are intimately associated and exhibit a dependency on the other cell type for their proper development; however, the mechanisms that underlie the functional relationship between macrophages and Leydig cells are unclear. Beyond the testis, in certain instances immune cells themselves, such as certain types of lymphocytes, are capable of steroid hormone production, thus highlighting the complexity and diversity that underlie steroidogenesis. In this review we will describe how immune cells are critical regulators of steroidogenesis in the testis and in extra-glandular locations, as well as discuss how this area of research offers opportunities to uncover new insights into steroid hormone production.

## Introduction

Steroid hormones are mainly produced in the adrenal glands, gonads, and placenta, where they play endocrine roles in regulating target tissue or cell function depending on circulating steroid concentrations (1, 2). While specific hormone-producing cells in these tissues have received the major share of focus in the field, previous studies have shown that many peripheral tissues and cell types within the brain, kidney, lung, skeletal muscle, intestine, keratinocytes, adipocytes, astrocytes, and placental trophoblasts have the capacity of *de novo* steroidogenesis or steroid conversion (3–11). This diversity of tissues with steroidogenic capacity indicates that there are multiple cell types that can undertake or mediate steroid hormone production. One cell lineage that has been linked to steroidogenesis is the immune cell lineage, as local sex steroid production has been identified within immune cell populations such as macrophages and T lymphocytes (12–14). Within the testis, macrophages have been implicated in steroid production by Leydig cells (15, 16), although the mechanisms by which macrophages developmentally or functionally regulate Leydig cells are poorly understood. The unexpected and poorly understood steroidogenic capacity of immune cells and their roles in modulating glandular steroidogenesis is becoming an emerging area of research that is critical for a deeper understanding of the complex immunoregulatory roles of steroid hormones in normal and disease contexts. In this review we will discuss the various roles proposed for testicular macrophages in Leydig cell biology and we will highlight future areas of research that should be pursued to elucidate the mechanisms underlying regulatory functions of immune cells and their potential *de novo* steroidogenesis in the testis and, potentially, beyond.

## Biosynthetic Pathway and Site of Production of Steroid Hormones

Steroidogenesis is a process in which cholesterol is converted into steroid hormones by a series of steps mediated by steroidogenic enzymes. In this process, there are two key rate-limiting steps, which are 1) the transport of cholesterol from the cytoplasm into mitochondria and 2) the conversion of cholesterol into pregnenolone. Free cholesterol is derived from intracellular cholesterol that is synthesized either from acetate, from cholesterol ester stored in lipid droplets, or from uptake of cholesterol-containing low-density lipoproteins (LDLs). Plasma LDLs are the most important source of cholesterol when steroidogenic cells are chronically stimulated. Then steroidogenic acute regulatory protein (StAR) promotes the rapid flux of cholesterol into the mitochondria, where cholesterol is catalyzed to yield pregnenolone by side-chain cleavage enzyme cytochrome P450scc (also known as CYP11A1, encoded by the *CYP11A1* gene) within the mitochondrial inner membrane. Pregnenolone, as an immediate precursor, requires further catalysis by two major families of enzymes, which are cytochrome P450 (CYP) and hydroxysteroid dehydrogenase (HSD) located in both mitochondria and the endoplasmic reticulum, to facilitate the biosynthesis of steroid hormones (2, 17, 18).

In many contexts, steroid hormones are classified based on the organs that produce them and the receptors to which they bind. The adrenal steroids, which consist of glucocorticoids and mineralocorticoids, are secreted by the adrenal cortex. Glucocorticoids such as cortisol in humans and corticosterone in rodents control many cell metabolic processes, including maintaining blood pressure and regulating immune cell function. Aldosterone is the most well-known mineralocorticoid, which maintains the body’s water and salt balance by acting primarily on the kidneys. Sex steroid hormones, which are composed of androgens (e.g., testosterone), estrogens (e.g., estradiol), and progestogens (e.g., progesterone), are produced by the gonads and placenta. These sex hormones are responsible for regulating sexual development and promoting fertility. Additionally, the adrenal cortex secretes sex hormones to a lesser extent than the gonads, and the gonads may produce adrenal steroids (1, 19). Aside from dedicated steroidogenic cells like Leydig cells, theca cells, or adrenocortical cells, future research should address the extent to which alternative glandular or extra-glandular cell types in the gonads and adrenal are involved in *de novo* steroidogenesis.

## Developmental Links Between Testicular Macrophages and Leydig Cells

Early analyses of the immune cells in the testis revealed that macrophages are a large component of the testicular interstitial compartment, comprising approximately 20% of interstitial cells (20). Macrophages and Leydig cells, therefore, occupy the same compartment of the testis and are in intimate contact throughout development (21). Histological and ultrastructural studies of the postnatal and adult rat testis demonstrated that macrophages and Leydig cells form intercellular cytoplasmic digitations (21, 22), which only are observed between these 2 cell types and only upon puberty (22), indicating an intimate relationship linked to testicular maturation. Furthermore, macrophage-deficient osteopetrotic mice mutant for *colony stimulating factor 1* (*Csf1^op/op^*) are infertile as a result of low testosterone, oligozoospermia, and decreased libido (15, 23, 24). Analyses of normal and cryptorchid testes revealed that there is a robust correlation between the volume density of Leydig cells and macrophages, as well as total mass of Leydig cells and macrophages per testis (25), leading to early ideas of functional coupling between the two cell types. These findings strongly suggest that testicular macrophages have trophic functions in Leydig cell differentiation and promote steroidogenesis, but the developmental and functional links between macrophages and Leydig cells are still open areas of investigation.

Multiple studies by Gaytan et al. in the 1990s revealed that there is an interdependent relationship between macrophages and Leydig cells in both developmental and regenerative contexts (26–28). Using dichloromethylene diphosphonate-containing liposome (Cl_2_MDP-lp) injection to deplete testicular macrophages in prepubertal rats, they found that macrophages are required for the development of Leydig cells during postnatal testicular maturation (26). The authors concluded that, in the absence of macrophages, Leydig cell proliferation did not occur, nor were mesenchymal progenitor cells able to undergo differentiation into Leydig cells (26). They further speculated that macrophages were required for Leydig cell responsiveness to lutenizing hormone (LH) and human chorionic gonadotropin (hCG) (29, 30), as hCG-treated Leydig cells in Cl_2_MDP-lp-injected testes did not increase in number as in contralateral intact testes. Regeneration of Leydig cells in testes that had selective Leydig cell depletion induced by ethylene dimethanesulfonate (EDS) treatment, which requires LH (31), was also hindered in the absence of macrophages (27, 28) (see next paragraph). These findings suggest that as-of-yet undefined macrophage factors are essential for Leydig cell responsiveness to LH/hCG.

Gaytan et al. demonstrated, again using a Cl_2_MDP-lp-mediated ablation method (27, 28), that testicular macrophages are required for adult Leydig cell regeneration after specific depletion of Leydig cells *via* EDS treatment. In contrast, when macrophages were ablated in intact adult testes, there was no effect on Leydig cell numbers (28), indicating that macrophages are not as essential for steady-state maintenance of adult Leydig cell numbers; a more recent finding showed a similar result, in which a diphtheria-toxin-mediated ablation of adult macrophages did not result in a change in Leydig cell number (although there was a significant drop in testicular testosterone levels) (32).

## Functional Relationship Between Testicular Macrophages and Leydig Cells

Given the tight physical association between testicular macrophages and Leydig cells in the interstitial compartment, in the past 40 years most investigations into testicular macrophage functions focused on Leydig cell steroidogenesis (16, 33). Yee and Hutson in 1985 showed that testicular macrophage-conditioned medium (TMCM) in a dose-dependent manner increases testosterone production of Leydig cells (34). Consistent with this finding, bank vole Leydig cells from a long photoperiod in co-cultures with testicular macrophages or treated with TMCM produced more testosterone (35). However, some subsequent studies demonstrated that non-stimulated testicular macrophages have an inhibitory effect on the production of testosterone by Leydig cells (36–38), whereas TMCM obtained from lipopolysaccharide (LPS)-stimulated macrophages or macrophages isolated from autoimmune orchitis could promote testosterone production (36, 39). Therefore, the role of testicular macrophages in Leydig cell steroidogenesis under physiological conditions has been controversial. Furthermore, testicular macrophages isolated using different methods may have different phenotypes and metabolic properties *in vitro* due to the loss of their complex *in vivo* microenvironment. This could be one of the reasons why testicular macrophages need to be additionally activated in some circumstances in order to function properly. Our recent study found that the depletion of adult testicular macrophages *in vivo* decreases testicular testosterone levels (32), suggesting the beneficial effect of testicular macrophages on Leydig cell steroidogenesis.

### Role of Testicular Macrophage-Derived Cytokines in Leydig Cell Steroidogenesis

A number of studies have shown that testicular macrophages from rats and goldfish can secrete pro-inflammatory cytokines, such as interleukin 1 (IL1) and tumor necrosis factor (TNF), which were dramatically increased after stimulation by LPS (40–42). Therefore, these cytokines from testicular macrophages may be key regulators of testosterone production, either enhancing or inhibiting it under physiological and inflammatory conditions. Previous research on the roles of IL1 on Leydig cell steroidogenesis *in vitro* yielded contradictory results. Many studies have shown that IL1B decreases testosterone synthesis of Leydig cells (43–45), whereas some studies reported that IL1B had no effects on testosterone synthesis of Leydig cells (37, 46), or even increased testosterone synthesis (47). Different testicular IL1 isoforms, including 17K IL1A and IL1B, 32K proIL1A, and a 24K splice variant, stimulated testosterone production by Leydig cells from 40- but not 80-day-old rats, and the potency of IL1A was 50-fold more than IL-1B (48). Intratesticular administration of IL1B resulted in a significant increase in basal testosterone secretion *in vitro* and serum testosterone concentration one day after treatment in 21-day-old rats, but it inhibited this process 6 days after treatment (49). A recent study showed that IL1B deficiency induced by treatment with diacerein, an anti-inflammatory agent, impairs Leydig cell function, suggesting a positive effect of IL1B in steroidogenesis under normal conditions (50). These findings suggest that the paracrine roles of IL1 in regulating Leydig cell steroidogenesis may be related to animal age, treatment time, and IL1 isoforms. Generally, numerous studies documented that TNF reduces testosterone production of Leydig cell function *in vitro* and *in vivo*. TNF treatment inhibited steroidogenic enzyme activity or their mRNA expression, such as StAR, CYP17A1, and HSD3B1, in a dose-dependent manner (51–55). Additionally, under LPS stimulation, testicular macrophages also could produce reactive oxygen species (ROS) and nitric oxide (NO) (33). Leydig cell steroidogenesis was inhibited by both hydrogen peroxide (a potent oxidant) (56, 57) and NO (58, 59). These results suggest that under inflammatory conditions, activated testicular macrophages secrete several factors that limit Leydig cell steroidogenesis and even impair testicular function.

Several groups’ studies have clearly demonstrated that there are two distinct macrophage populations in adult testis: 1) interstitial macrophages located in the testicular interstitium and in close contact with Leydig cells; and 2) peritubular macrophages located in the myoid layer around seminiferous tubules (32, 60–65). Interstitial macrophages express higher levels of the immunosuppressive M2-type gene *Il10*, while peritubular macrophages highly express the M1-associated inflammatory gene *Il1b* (62). However, whether IL10 and IL1B can be secreted into the testicular interstitial compartment by the two macrophage populations and whether the two populations have unique or overlapping roles in regulating Leydig cell steroidogenesis have been not investigated.

### Role of Testicular Macrophage-Derived Lipophilic Factors in Adult Leydig Cell Steroidogenesis

Aside from cytokines, a testicular macrophage-derived factor implicated in steroidogenesis was a lipophilic factor later identified as 25-hydroxycholesterol (25-HC) after it was purified using organic extraction and high-performance liquid chromatography (66, 67). Furthermore, human macrophages have been shown to produce 25-HC, indicating that this phenomenon is not specific to rodents (68). 25-HC is an oxysterol that is synthesized from cholesterol by the addition of a hydroxyl group to the position 25 carbon, and this reaction is catalyzed by cholesterol 25-hydroxylase (CH25H) (69). CH25H is found in the endoplasmic reticulum and is widely expressed in many cell types, particularly macrophages (70). The intracellular level of 25-HC is primarily determined by the activity of CH25H, which is upregulated *via* TLR4/IRF3/IFN-β/STAT1 signaling pathways in LPS-stimulated macrophages (71).

Recent studies have found that macrophages have the potential to provide an alternative pathway for steroidogenesis by providing 25-HC as a direct substrate for side chain cleavage (16, 72). 25-HC has been shown to increase StAR protein levels in Leydig cells and adrenocortical cells *in vitro* (73). Kazeto et al. transfected non-steroidogenic cells with a complex of eel *P450scc* cDNA (encoding *Cyp11a1*) and discovered that the recombinant CYP11A1 produced in these cells efficiently catalyzed the conversion of 25-HC into pregnenolone (74). A recent study revealed that Leydig cells utilize 25-HC as a substrate for testosterone biosynthesis (72), in which it was proposed that cholesterol is converted into 25-HC by CH25H in macrophages, and the 25-HC is subsequently secreted into neighboring Leydig cells. In Leydig cells, StAR transports 25-HC to mitochondria where is converted into pregnenolone by the CYP11A1 enzyme. 25-HC produced in macrophages promotes testosterone synthesis in Leydig cells, while testosterone produced in Leydig cells inhibits 25-HC production in macrophages (75), which suggests a paracrine negative feedback loop between the two cell types. Therefore, 25-HC could be a paracrine factor that mediates interactions between macrophages and neighboring Leydig cells.

## Steroid Production by Immune Cells

Tissue immune cells, particularly macrophages and T lymphocytes, may be an important source of local steroid production by steroid conversion or *de novo* steroidogenesis. Intracrine and paracrine roles of immune-cell-derived steroids may be essential for cellular functions within various tissues. Therefore, immune cell-derived steroids and steroid metabolites potentially have biological effects within the tissue microenvironment, although their quantities in tissue fluids or blood are likely modest.

### Steroid Conversion Capacity of Immune Cells

Immune cells are not only passive targets of steroid hormones due to their expression of hormone receptors, but also have the capacity for steroid hormone conversion and metabolism (14). Human alveolar macrophages can convert androstenedione to testosterone and other steroids through the catalytic activity of 3β-HSD, 3α-HSD, 17β-HSD, and 5α-reductase enzymes (76). These steroidogenic enzymes also are present in the alveolar macrophages of pigs (77), indicating an evolutionary conservation of these steroidogenic functions. In turn, testosterone is converted to androstenedione and dihydrotestosterone (DHT) in primary cultured human synovial macrophages (78, 79). In addition, human monocyte-derived macrophages, rather than monocytes, preferentially convert dehydroepiandrosterone (DHEA) to a physiologically relevant amount of downstream steroid hormones including testosterone, androstenedione, estrone, and estradiol, in the presence of LPS (80). When human peripheral monocyte-derived THP-1 cells and primary monocytes are differentiated to macrophages, they exhibit upregulation of both *CYP19A1* mRNA levels and aromatase activity, which catalyzes the conversion of androgens to estrogens, in response to dexamethasone (a synthetic glucocorticoid) (81). These studies suggest that the conversion of steroid hormones in macrophages may be related to their phenotypic heterogeneity and microenvironmental contexts.

Steroidogenic enzymes are also expressed by T lymphocytes. Splenic T lymphocytes in trauma-hemorrhagic male and proestrus female mice exhibited enzyme activities of 3β-HSD, 17β-HSD, 5α-reductase, and aromatase (CYP19A1). Although most of these steroidogenic enzymes were also found in B lymphocytes, they had lower activity and no 17β-HSD activity. Increased 5α-reductase activity in male T cells is immunosuppressive due to enhanced 5α-dihydrotestosterone synthesis, whereas increased aromatase activity, which triggered 17β-estradiol synthesis, has an immune-protective function in female T cells (82). Furthermore, *CYP19A1* expression and aromatase activity has been reported in tumor-infiltrating lymphocytes (83, 84). However, whether other lymphocytes and/or myeloid cell types in normal tissues have steroidogenic activities that can induce the conversion of steroid hormones to fulfill their immunoregulatory functions is likely a fruitful area for future research.

### *De Novo* Steroidogenesis of Immune Cells

Beyond immune cells’ capability of local steroid conversion, recent reports indicate that immune cells have the ability to undertake *de novo* steroidogenesis starting from the initial processing of cholesterol. Type 2 immune cells, including mast cells, basophils, and particularly T helper 2 cells, can *de novo* synthesize pregnenolone during helminth infection and in tumor environments to regulate immune homeostasis and tumor immunosuppression, respectively. T-helper-2-cell-mediated steroidogenesis is likely due to the high expression of CYP11A1 in these immune cells (12, 13). CYP11A1 expression is increased in CD4+ or CD8+ T cells in peanut-induced intestinal anaphylaxis and allergic lung disease (85, 86). Additionally, in peanut-allergic children, CYP11A1 is involved in the regulation of CD4+ T cells in the proallergic immune response (87). These findings may suggest the importance of steroids derived from immune-cell-mediated *de novo* steroidogenesis in healthy and pathological microenvironments with adaptive immunomodulation. In addition, infiltrating myeloid cells in dystrophic skeletal muscles can produce aldosterone, as all genes encoding steroidogenic enzymes in the aldosterone synthesis pathway are expressed by muscle-derived myeloid cells (88). However, whether tissue-resident or inflammation-induced macrophages are capable of *de novo* steroidogenesis has yet to be determined. StAR has been detected in macrophages (89, 90), indicating that macrophages contain at least the ability to produce steroidogenic substrates. Interestingly, primary testicular macrophages produce significant amounts of corticosterone *in vitro* (91), but whether this corticosterone is derived from the conversion of other steroids or from *de novo* steroidogenesis was not investigated in that study. A recent study reported that testicular macrophages could also produce progesterone, and this steroid production by macrophages may contribute to a local feedback loop between Leydig cells and macrophages that regulates testosterone production (92). Therefore, it is necessary to explore in greater detail whether and how testicular macrophages have the ability to undertake *de novo* steroidogenesis and, if so, to what extent testicular function is dependent on this source of steroidogenesis.

## Discussion

**Figure 1:**
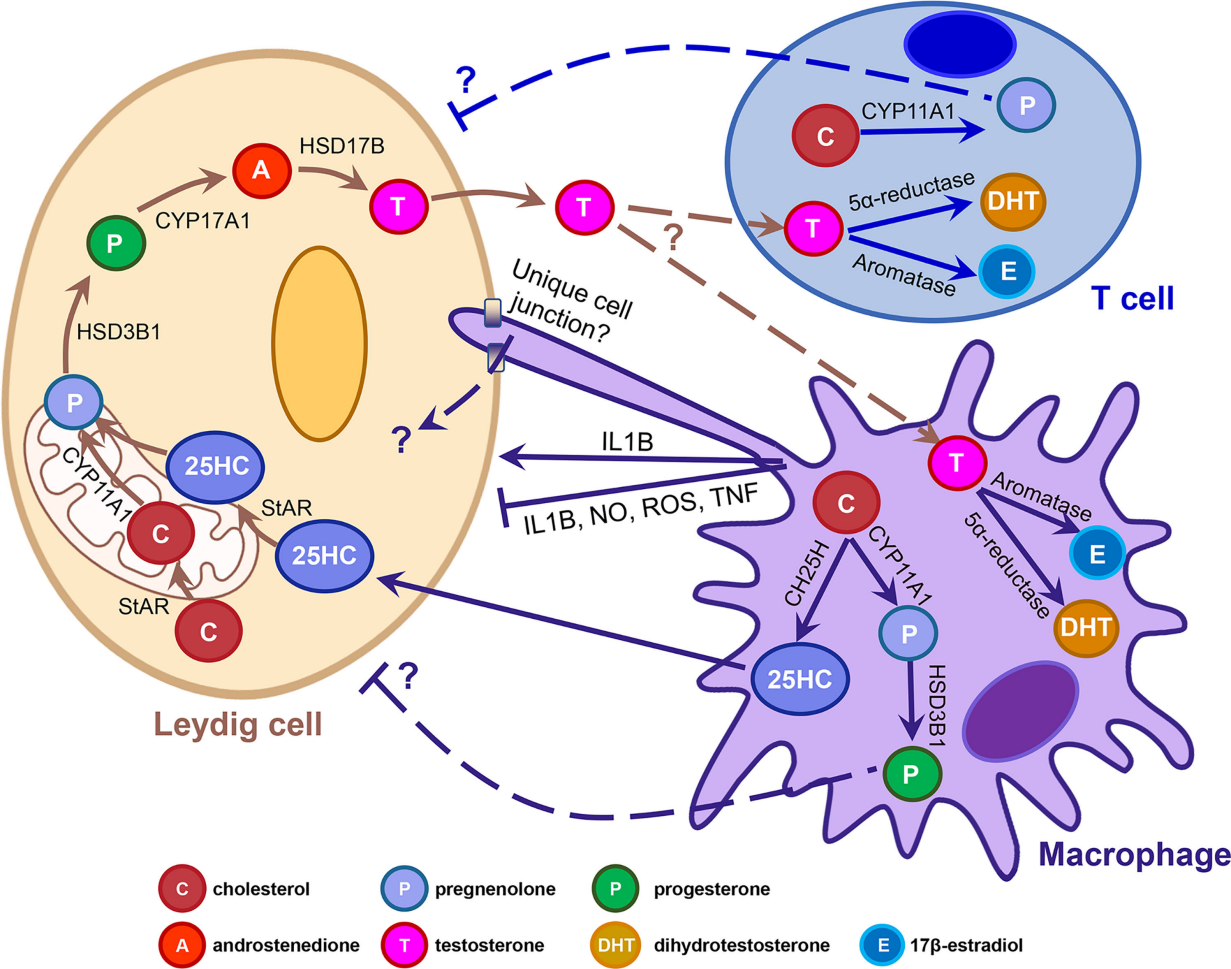
Potential mechanisms underlying macrophage-Leydig cell interactions and immune cell steroidogenesis. Cartoon depicts the adult rodent testicular interstitium, containing a Leydig cell, macrophage, and T cell. Arrows denote the different molecular and cellular pathways that have been implicated in macrophage-Leydig interactions and *de novo* steroidogenesis by immune cells. T-shaped lines indicate an inhibitory interaction. Dashed arrows and lines flanked by question marks indicate that interactions have been proposed but have not been demonstrated experimentally, nor have mechanisms or factors involved been identified definitively. 25HC, 25-hydroxycholesterol; CH25H, cholesterol 25-hydroxylase; IL1B, interleukin 1 beta; NO, nitric oxide; ROS, reactive oxygen species; StAR, steroidogenic acute regulatory protein; TNF, tumor necrosis factor.

The presence of testicular macrophages and their potential roles in Leydig cell steroidogenesis have been investigated for several decades, but the mechanisms underlying their functional relationship is still unclear. One particular area that needs to be rigorously addressed is whether testicular macrophages merely promote steroidogenesis by Leydig cells or if they undergo *de novo* steroidogenesis in a meaningful way to promote spermatogenesis and fertility. Macrophages could impact Leydig cells through a number of mechanisms, such as regulating the cytokine environment, providing steroidogenic substrates, or through modulating Leydig cell ultrastructure *via* unique cell-cell junctions (**Figure 1**). Given recent findings of *de novo* steroidogenesis by T cells in various contexts, the contributions of immune-cell-derived steroids should be addressed in the context of testicular function. Furthermore, as many studies have linked inflammation to infertility, it is also critical to study how macrophage polarization and the subsequent changes in their cellular activities cause or exacerbate testicular pathology. Reports in several fields indicate that immune cell steroid production is a broadly observed and evolutionarily conserved phenomenon; therefore, understanding the roles of immune cells in testicular steroidogenesis and Leydig cell function will likely provide new insights into endocrinology that will extend beyond the boundaries of the testis.

## Author Contributions

XG, S-YL, and SM performed literature searches and drafted the manuscript. TD conceptualized, drafted, and supervised the manuscript. All authors contributed to manuscript revision and approved the submitted version.

## Funding

Work from the DeFalco laboratory is supported by National Institutes of Health (grants R35GM119458 and R01HD094698 to TD).

## Conflict of Interest

The authors declare that the research was conducted in the absence of any commercial or financial relationships that could be construed as a potential conflict of interest.

## Publisher’s Note

All claims expressed in this article are solely those of the authors and do not necessarily represent those of their affiliated organizations, or those of the publisher, the editors and the reviewers. Any product that may be evaluated in this article, or claim that may be made by its manufacturer, is not guaranteed or endorsed by the publisher.
